# PARP Inhibitors and Proteins Interacting with SLX4

**DOI:** 10.3390/cancers15030997

**Published:** 2023-02-03

**Authors:** Lars Petter Jordheim

**Affiliations:** Univ Lyon, Université Claude Bernard Lyon 1, INSERM 1052, CNRS 5286, Centre Léon Bérard, Centre de Recherche en Cancérologie de Lyon, 69008 Lyon, France; lars-petter.jordheim@univ-lyon1.fr

**Keywords:** olaparib, DNA repair, SLX4, cancer, treatment

## Abstract

**Simple Summary:**

The aim of this review is to highlight recent work performed on the activity of a class of cancer drugs (PARP inhibitor) in various cell lines, either expressing or not expressing proteins interacting with the DNA repair protein SLX4. This could be at the basis of the development of new therapeutic strategies in cancer treatment.

**Abstract:**

PARP inhibitors are small molecules currently used with success in the treatment of certain cancer patients. Their action was first shown to be specific to cells with DNA repair deficiencies, such as BRCA-mutant cancers. However, recent work has suggested clinical interest of these drugs beyond this group of patients. Preclinical data on relationships between the activity of PARP inhibitors and other proteins involved in DNA repair exist, and this review will only highlight findings on the SLX4 protein and its interacting protein partners. As suggested from these available data and depending on further validations, new treatment strategies could be developed in order to broaden the use for PARP inhibitors in cancer patients.

## 1. Introduction

The PARP-1 protein is involved in single-strand break repair by recruiting XRCC1 to the site of modified DNA. When PARP is inhibited, cells rely on other DNA repair mechanisms, particularly homologous recombination, to correctly replicate genomic information without the risk of undergoing lethal mitoses. In cells deficient for homologous recombination, such as BRCA1- or BRCA2-mutated cells, PARP inhibition is lethal [1,2]. After these descriptions were provided in 2005, the concept of synthetic lethality was born and PARP inhibitors (PARPi) were developed for the purpose of treating BRCA-mutated patients in which non-cancerous cells have one wild-type allele, whereas cancer cells are BRCA deficient and thus specifically sensitive to PARPi. Several PARPi have been clinically studied and are available for the treatment of cancer patients (olaparib, rucaparib, talazoparib, niraparib and veliparib (ABT-888)). The scientific literature on PARPi is overwhelmingly rich (>12,000 papers since 2005) with research papers, clinical trials and reviews emerging about the mechanisms of action, resistance, clinical activities and the development of new compounds.

The mechanism of action of PARPi was initially thought to be a “simple” inhibition of PARP1-related single-strand break repair and the subsequent appearance of much more toxic and less easily reparable double-strand breaks. However, the truth is much more complicated, as discussed already more than a decade ago by T. Helleday [3] and extensively since that publication [4]. Work based on this knowledge facilitated the identification of other proteins involved in the mechanism of PARPi activity and resistance, and aided in the development of association strategies with pharmacological inhibition of other DNA-repair-related proteins such as RAD51 [5] and EZH2 [6].

Much is still unknown about PARPi and they probably have a stronger clinical potential than described today. In particular, the possible involvement of other DNA repair mechanisms and associated proteins in PARPi activity would be of interest in the development of new therapeutic strategies based on patient selection or drug associations.

**Figure 1:**
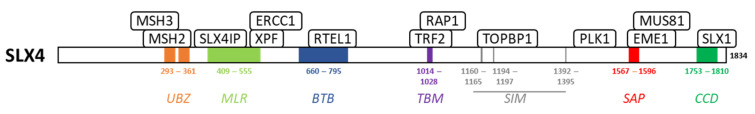
Schematic structure of SLX4 protein with different protein domains (in colors) and interacting proteins. Adapted from [9]. BTB: Bric-a-brac, tramtrack, broad complex; CCD: Conserved C-terminal Domain; MLR: MUS312-MEI9 interaction-like region; SAP: SAF-A/B, Acinus and PAIS; SIM: SUMO-interacting motif; TBM: TRF2-binding motif; UBZ: ubiquitin-binding zinc finger type 4.

SLX4 (FANCP, BTBD12) is a DNA repair protein that has gained increasing interest over the last decade. It is a tumor suppressor whose deficiency causes Fanconi anemia, and it is involved in genomic stability through several DNA-related processes (recombination, replication fork restart, telomere maintenance, interstrand crosslink repair) [7,8]. Indeed, SLX4 works as an interaction scaffold or protein hub, directly interacting with several important DNA repair proteins (Figure 1). Some of these proteins are involved in the sensitivity of cancer treatments such as MSH2 for 6-thioguanine and XPF for cisplatin and mitomycine C (MMC). The physical interactions between these proteins and SLX4 are expected to regulate protein activities by stimulating or inhibiting them. In the initial work on SLX4, including cell models expressing different variants of SLX4, the sensitivity to cancer is often used as a read-out to evaluate the implications of the studied interactions in different DNA repair pathways. This has shown that SLX4 and/or certain protein interactors of SLX4 can modulate the cellular response to cancer treatments. Given the involvement of SLX4 in homologous recombination and its potential regulation of interacting proteins, the mentioned studies sometimes include the sensitivity to PARPi in the characterization of cell models. These studies sometimes suggest the role of these proteins in the activity of PARPi, but no extensive work has yet been published to conclude on this.

The purpose of this review is not to detail various DNA repair mechanisms and the role of each protein therein, nor to describe the mechanisms of action and resistance of PARPi, but to present and discuss work on SLX4 and proteins directly interacting with this protein and their potential involvement in the activity and cellular response to PARPi. The choice of SLX4 is based on its involvement in various DNA repair mechanisms and the important number of interacting proteins, as well as its relatively recent identification in humans. This oncopharmacological approach will facilitate the identification of interesting approaches that can be further developed in order to broaden the clinical use of PARPi. It is complementary to what can be presented about other DNA-repair proteins, and to a mechanistic approach that would identify the molecular mechanism of action and explain the sensitivity to PARPi in such cell models. Published data are reviewed protein by protein. For some SLX4-interacting proteins, there is no currently available information on their potential role in PARPi sensitivity.

## 2. SLX4

When SLX4 is recruited to laser-induced DNA damage, it interacts with PARP1 and is PARylated [10]. This is slowed down by PARPi, suggesting a functional interaction between these two proteins, and thus a role of SLX4 in PARPi sensitivity.

A non-cancerous SLX4-deficient cell line (RA3331) derived from an FANCP-patient was transfected with variants of SLX4 in order to study the effect of this protein and its different domains in DNA repair and in sensitivity to drugs [11]. The introduction of wild-type SLX4 in these cells induced a more than 100-fold increase in the IC50 to KU58948 (an olaparib-derivative, >1000 nM vs. 10 nM) showing that SLX4 deficiency was associated with a higher sensitivity to PARPi. Cells expressing proteins without an interaction with the EME1/MUS81 complex (SAP domain) were as sensitive to PARPi as SLX4-deficient cells, whereas the deletion of the CCD domain (interaction with SLX1) produced an intermediate phenotype (IC50: 1000 nM). The deletion of the domain for interaction with XPF/ERCC1 or mutations in the domain for interaction with MSH2/MSH3 did not induce modifications in sensitivity to PARPi as compared to the wild-type protein, suggesting that these interactions are not needed for the underlying mechanism of PARPi sensitivity. This was later confirmed by work performed on the same cells in another laboratory [12]. The results obtained with these cells, which were transfected with clinically relevant SLX4 variants (SLX4 mutations identified in hereditary breast cancers), showed that certain missense mutations did not notably affect the sensitivity to olaparib (IC50: >1000 nM), whereas cells expressing a truncated SLX4 (after W823, missing TBM, SIM, SAP and CCD) were as sensitive to olaparib as SLX4-deficient cells (IC50: 50 nM) [13]. This again confirmed the initial observations on these cells. Recently, an SLX4-knock-out HeLa cell model was developed with CRISPR-Cas9 technology [14]. The cells produced a shorter N-terminal truncated form of SLX4 (360–1834) due to an alternative translation initiation site. These cells were hypersensitive to MMC (approximately 5% vs. 95% survival at 1 ng/mL), but unfortunately were not evaluated for PARPi sensitivity. This variant lost interaction with MSH2 (by the loss of the newly identified SHIP box upstream of the UBZ domain) but retained interactions with other partners. Additional work on SLX4 variants was performed on models from murine embryonic stem cells [15]. The expression of only one copy without the interacting domain with MUS81 (SAP), with SLX1 (CCD) or with XPF (MLR) or the domain for recruitment to damaged DNA (UBZ), did not significantly increase the sensitivity to olaparib as compared to cells expressing one copy of wild-type protein (IC50 always >100 nM). Even though human and murine proteins have some differences, their structures, particularly concerning the described domains for interactions, are similar overall. Therefore, these results suggest a specificity within the species or eventually within tissues; however, some qualitative and quantitative SLX4 variations are associated with PARPi activity.

## 3. MSH2/MSH3

The colon cancer cell line HCT-116 is deficient in mismatch repair due to loss of chromosomes 3 (MLH1) and 5 (MSH3), but cell lines in which these chromosomes have been introduced exist. Cell-based in vitro experiments on such HCT-116 models showed that cells expressing the protein were less sensitive to cisplatin (approximately 50 vs. 20% survival at 5 µM) and oxaliplatin (approximately 60 vs. 40% survival at 2 µM) as well as to olaparib (approximately 35 vs. 20% survival at 2 µM) as compared to MSH3-deficient cells [16]. By exposing human breast cancer cells (MDA-MB-231) to the growth factor TGF-β, the sensitivity to veliparib was increased (65 vs. 80% survival at 60 nM) [17]. Although other modifications most likely occurred, this was shown to be associated with, and probably due to, a 75% decrease in MSH2 expression. Indeed, when MSH2 was overexpressed by transfection in these same cells, the sensitivity to PARPi decreased (100 vs. 40% survival at 10 µM), even though the siRNA-mediated inhibition of MSH2 only induced a very moderate sensitization to these drugs. In a work on homologous recombination and the proteins MLH3 and PMS2 in human B cells (TK6), knock-down of MSH2 expression was obtained with CRISPR/Cas9 technology [18]. The corresponding cells had increased sensitivity to the alkylating agent temozolomide (10 vs. 100% survival at 100 µM), but were equally sensitive as control cells to 400 nM olaparib and 2 nM camptothecin. Thus, as with SLX4, there might be tissue specificity for the role of MSH2/MSH3 in the cellular response to PARPi, showing sensitization in deficient colon and breast cancer cells, but not in lymphocytes.

## 4. ERCC1/XPF

The role of this heterodimer in the sensitivity to alkylating agents is well documented, whereas there is much less work reported on PARPi. Veliparib showed enhanced activity when combined with carboplatin in BRCA1- and BRCA2-deficient and -proficient murine fibroblasts and human cancer cells (HCC1973 and EUFA423F) [19]. Surprisingly, the association with cisplatin was rather antagonistic. As for other cells, the expression of ERCC1 in these BRCA cells was correlated with sensitivity to platinum derivatives, but whether this is the case for the PARPi-carboplatin association is not clear. In another study, veliparib and olaparib were highly active on cells with a low expression of ERCC1 (HCC827 (20% survival at 3 µM olaparib, 10% survival at 50 µM veliparib) and PC9 (10% survival at 3 µM olaparib, 20% survival at 10 µM veliparib)), but less on cells with high ERCC1 expression (A549 (50% survival at 3 µM olaparib, 20% survival at 50 µM veliparib) and H157 (50% survival at 3 µM olaparib, 20% survival at 50 µM veliparib)) [20]. However, as different cell lines were used, other factors could be involved in this PARPi sensitivity. In the same study, an siRNA-mediated knock-down of ERCC1 was associated with an increased sensitivity to PARPi and to cisplatin in A549 (70 vs. 100% survival at 1 µM for olaparib, 50 vs. 100% survival at 1 µM for cisplatin) and H157 cells (50 vs. 70% survival at 1 µM for olaparib, 20 vs. 30% survival at 1 µM for cisplatin). Additionally, the association between PARPi and cisplatin was shown to be synergistic. Later, it was shown that both cisplatin (IC50: approximatively 10 nM vs. 10 µM) and PARPi (Olaparib—IC50: approximatively 0.1 µM vs. 10 µM and niraparib) were more active in ERCC1-deficient lung cancer cells (A549) as compared to their ERCC1-expressing controls, although the difference was lower than that between BRCA2-positive (IC50: 1 µM) and BRCA2-negative cells (IC50: approximately 20 nM) [21]. Sensitization to PARPi was also obtained in a short-term assay with siRNA-mediated inhibition of ERCC1 expression (IC50: 1 µM vs. 0.03 µM). A work on different triple-negative breast cancer cell lines with various BRCA status and PARP1 expression levels showed that there was no evident association between veliparib sensitivity and ERCC1 expression level [22].

When PARPi inhibits PARP1 and modifies subsequent DNA repair in ERCC1-deficient and BRCA1-mutant breast cancer cells, chromatin fragments are released into the cytoplasm and trigger immunomodulatory pathways (cGAS/STING) [23]. This effect is dependent on type I interferon, and PARPi can also increase PD-L1 expression in NSCLC cells via the same mechanism. The latter is increased in ERCC1-deficient cells and can aid in the development of PARPi-based combination therapies in lung cancer [24], as suggested earlier for ovarian cancer [25]. Olaparib-resistant cell lines for gastric cancer (SNU-484, SNU-601, SNU-668 and KATO-III), obtained by long-term exposure to the drug, showed modifications in the ATM/CHK2 pathway as well as either an increase of 1.4–3.7-fold (3 cell lines) or decrease of 3.6-fold (1 cell line) in ERCC1 expression [26]. The cells with decreased ERCC1 expression had a slightly increased sensitivity to both cisplatin and camptothecin. In lung cancer cells (NCI-H1299 and SK-MES-1) with increased or decreased ERCC1 expression, the sensitivity to a combination of cisplatin and olaparib was decreased when ERCC1 was overexpressed (50 vs. 68% survival in NCI-H1299, 11 vs. 17% survival in SK-MES-1 cells) and increased when ERCC1 was downregulated (12 vs. 6% survival in NCI-H1299, 28 vs. 9% survival in SK-MES-1 cells) [27]. However, no data were available on olaparib alone in this study.

Lymphocytes from individuals with mutations in the ERCC4 gene (coding XPF or FANCQ protein) are, as expected, more sensitive to MMC (IC50: 20–40 nM vs. 300 nM), but do not have modified sensitivity to KU58948 [28]. The inhibition of either ERCC1 or XPF expression by siRNA in p53 wt (A2780, 3–6% vs. 20% survival at 10 µM) and p53-mutated (PEO4, <1% vs. 80% survival at 2 µM) ovarian cancer cells increased the sensitivity to olaparib [29]. This was also the case in cisplatin-resistant A2780 cells with an increased expression of both ERCC1 and XPF (3–10% vs. 60% survival at 10 µM). However, the exogenic expression of XPF in MDA-MB-468 breast cancer cells did not modify their sensitivity to 100 µM olaparib, whereas it did decrease the sensitivity to 2.5 µM cisplatin (6 vs. 20% survival) [30]. Murine embryonic stem cells expressing only one copy of XPF did not have modified sensitivity to olaparib, whereas when the nuclease domain was deleted cells were slightly more sensitive to this PARPi (50 vs. 100% survival at 100 nM) [15].

Again, the results suggest that there are differences in the involvement of these proteins in the response to PARPi between tissues or cell models, and additional experiments (in vivo or on patient samples) would be needed to make conclusions.

## 5. TRF2/RAP1

TRF2 is recruited to DNA for telomerase-independent repair in a two-step mechanism [31]. The first step is PARP-dependent and is inhibited by PARPi, suggesting a mechanism for DNA-repair inhibition by these drugs. However, there is no knowledge on the eventual sensitivity of cells with modified TRF2 expression, as there are also no data on RAP1 and PARPi.

## 6. PLK1/TOPBP1

Polo-like kinase 1 regulates the cell cycle during mitosis but also participates in DNA damage. PLK1 is recruited to DNA double-strand breaks within a mechanism dependent on PARP1, as shown by the inhibition of the recruitment by PARPi [32]. Subsequently, PLK1 is phosphorylated by CHK1 and in turn phosphorylates the RAD51 that is needed for DNA repair.

When BRCA2-expressing and olaparib-resistant pancreatic cancer cells (CAPAN1-derived) were transfected to express Aurora-A (kinase involved in mitosis), cells were equally sensitive as untransfected BRCA2-deficient CAPAN1 cells to PARPi KU58948 in both in vitro (IC50: approximately 10 nM vs. 50 µM for untransfected cells) and in xenograft models [33]. This was supposed to be due to the inhibition of homologous recombination by the activation of PLK1 and subsequent inhibition of CHK1 upon Arora-A expression. The siRNA-mediated inhibition of topoisomerase-IIb-binding protein (TOPBP1) expression in human osteosarcoma U2OS cells was associated with an increase in the sensitivity to olaparib (approximately 10 vs. 100% survival at 1 µM) [34]. This was similar to the effect obtained by BRCA2 inhibition, and can explain the synergy seen between PARPi and topoisomerase-1 inhibitors such as camptothecin [22]. TOPBP1 inhibition is supposed to mimic PLK1 inhibition because TOPB1 interacts with PLK1 and increases the RAD51-phosphorylation needed for DNA repair [34].

In castration-resistant BRCA2-mutant prostate cancer cells (22RV1), olaparib was synergistic with the PLK1-inhibitor BI2536 [35]. This also helped to obtain therapeutic activity in a xenograft model that was not observed in BRCA2-positive cells (C4-2). Interestingly, PLK1 was increased in olaparib-exposed 224V-1 cells and decreased in similar conditions in C4-2 cells. Two gastric cancer cell lines (MKN45 and AGS) were sensitive to PARPi (olaparib) and a WEE1/PLK1 inhibitor (AZD1775), and the combination of olaparib/AZD1775 led to a much better response [36]. PLK1 was also identified as being synthetically lethal with BRCA1 and BRCA2, as is the case for PARPi [37]. A study conducted on two ovarian cancer cells, one being rather sensitive (OVSAHO) and one rather resistant (Kras-mutated KURAMOCHI) to olaparib, showed that the sensitivity to PLK1-inhibitor BI6727 was higher in KURAMOCHI than in OVSAHO cells [38]. Additionally, PARP expression increased more rapidly in resistant cells upon olaparib exposure. The number of γH2AX foci was increased by olaparib after MMS-exposure, and the association with BI6727 increased this even further. This also resulted in an increased sensitivity to the association of MMS/olaparib/BI6727 as compared to MMS with either of the other compounds.

PrimPol is a target for phosphorylation by PLK1 on serine 538, and when this phosphorylation is impossible due to the expression of an alanine-mutant, cells have an increased sensitivity to olaparib (1% vs. 40% survival at 1 µM) and camptothecin (10 vs. 40% survival at 5 nM) [39]. PLK1 also phosphorylates MRE11 on S649, and prostate cancer cells (U2OS) expressing phosphomimetic MRE11 have a 5-fold increased sensitivity to olaparib, both in vitro and in vivo [40]. The phosphorylation decreases the DNA binding of MRE11 and does not allow for the downstream ATM/Chk2-signaling that is needed for DNA repair. This is somewhat surprising, as the siRNA-mediated decrease in MRE11 expression is associated with an increased sensitivity to PARPi [41] and would suggest that patients with high PLK1 expression would benefit from PARPi-based treatment.

As seen here, the inhibition of PLK1 is most often associated with increased sensitivity to PARPi, even though its role in the phosphorylation of MRE11 is somewhat controversial.

## 7. MUS81/EME1

The siRNA-mediated inhibition of survivin in three human breast cancer cell lines (Cal51, MCF7 and MDA-MB-231) sensitized them to olaparib [42]. This was associated with a decrease in the expression of MUS81 and RAD51, which could explain this sensitization, although the direct involvement of MUS81 was not evident. The inhibition of MUS81 expression by shRNA in human ovarian cancer cells (A2870 and SKOV3) induced a mild sensitization to olaparib (IC50: 4 vs. 7 µM for A2870, 6 vs. 9 µM for SKOV3) that was associated with an increase in the expression of MCM2 [43]. In the same models, the inhibition of MCM2-expression decreased MUS81 expression, but there were no data on PARPi sensitivity in this condition. Similar sensitization was seen in another study on ovarian cancer (HO8910 and SKOV3) in which the shRNA-mediated inhibition of MUS81 expression was associated with a sensitization to olaparib and camptothecin, together with an increase in the expression of the protein BM28 that is involved in early steps of DNA replication [44]. In this study, the inhibition of MUS81 did not modify RAD51 expression, whereas the inhibition of RAD51 expression decreased MUS81 expression. When MUS81 expression was inhibited in human B cells (TK6), cells became slightly more sensitive to 200 nM olaparib and 2 nM camptothecin [18]. This was even more pronounced when the mismatch repair protein PMS2 was also inhibited. In gastric cancer cells (SGC7901 and BGC823) with shRNA-mediated downregulation of MUS81, the sensitivity to talazoparib was increased approximately 2-fold (IC50: 0.4 vs. >1 µM) in a 5-day MTS experiment, whereas the difference was more important in a 10-day clonogenicity assay (7–12% versus 65–72%) [45]. Better antitumoral activity was also observed in a xenograft in vivo model. MUS81 was both overexpressed and downregulated (shRNA) in castration-resistant prostate cancer cells (PC3 and DU145) [46]. Increased expression was associated with mildly increased cell proliferation and decreased sensitivity to olaparib, whereas in cells with decreased MUS81 expression the effect was the opposite. When MUS81 expression was decreased in HELA cells, the sensitivity to PARPi decreased in BRCA2-deficient cells but did not vary in BRCA1-deficient cells [47]. In this same study, it was shown that MUS81 expression was slightly lower in PARPi-resistant mouse tumors as compared to PARPi-sensitive tumors.

Olaparib resistance was induced in ovarian cancer cells (PEO1) either by a high initial dose and short exposure or a low initial dose and long exposure [48]. In the first case, cells reactivated BRCA2 and increased drug efflux through ABCB1 expression, whereas in the second case, cells expressed mesenchymal markers that were shown to be responsible for the resistance. In both models, MUS81 and EZH2 expressions were decreased, especially upon PARPi exposure. Finally, both models were highly sensitized to olaparib by siRNA targeting RAD51, in contrast to parental-sensitive cells.

Overall, it seems that inhibiting MUS81 is a suitable method for increasing the sensitivity of cancer cells to PARPi.

## 8. General Discussion and Perspectives

The previously published work that is reviewed here shows that some of the SLX4-interacting proteins are involved in the cellular response to PARPi. However, the conclusions of the various observations and the potential clinical applications are dependent on the experimental designs and on further experiments. For example, the potentiation of, or synergistic effect with, PARPi has been studied using either transient inhibition of protein expression (siRNA, shRNA), transient inhibition of protein activities (enzymatic inhibitors), or stable inhibition or activation of protein expression (CRISPR/Cas9 or transfections). The effect on the cell is not the same with the various protocols, as the studied protein is either present or absent. In the first case, even with enzymatic inhibitors, the protein can interact with other proteins and ensure non-enzymatic activities occur. This has been nicely evaluated, for example, in studies on SLX4, with cell models expressing variants with or without given domains. In the second case, cells with stable modifications have time to adapt to changes induced by the up- or downregulation of expression and to modify accordingly. These modifications can be related to false-negative results concerning the effect of PARPi, as they will probably not be observed with a pharmacological inhibitor and drug candidate. Therefore, any potential drug candidate should be validated with small inhibitors and not only with engineered cell models. Another limitation to some of the presented research is the use of non-cancer cells (fibroblasts, lymphocytes…). Cell biology, particularly the DNA repair capacities, cell cycle control and telomere maintenance of cells, is very often different between cells and cancer cells, underlining the need for confirmation of data in more relevant models. As already mentioned, conclusions can be even more difficult to make based on tissue or species specificities, and this also provides motivation for further studies, including studies of cell models of various origins. Further, the clinical relevance of the presented results will also depend on the expression pattern of the studied proteins in cancer patients, and more precisely on the differences in expression between cancer cells and healthy cells. This is indeed the basis of synthetic lethality, the concept within PARPi that was initially developed. However, the results discussed in this review also highlight that PARPi can be used in situations with only modified expressions and not necessarily deficiencies, and this can of course also be true for other proteins not included in this review. In addition, the association between a PARPi and an inhibitor of the discussed proteins is expected to be of therapeutic interest, even though the specificity for cancer cells will then be lost. As the proteins interact physically with SLX4, inhibitors of protein–protein interaction represent an alternative to classical enzyme inhibitors.

SLX4 was identified in humans in 2009 with a thorough description of interacting proteins and their role in DNA repair [49,50,51]. It has been further studied in a large series of papers and reviews [7,8,52] and associated with a group of patients with Fanconi anemia (subtype FA-P) [53,54]. Further studies on this and associated proteins, associated with in-depth knowledge of structures and regulating interactions, should facilitate the development of new treatment strategies based on alkylating agents, topoisomerase inhibitors or PARPi.

## 9. Conclusions

As indicated in the introduction, the mechanism or action of PARPi in BRCA-deficient cells is not as evident as initially imagined. To understand the mechanism of action in cells with a modified expression of SLX4-interacting proteins, extensive work is necessary. Indeed, whether the PARPi sensitivity is associated with more DNA damage, different DNA damage, telomerase instability, a facilitated cell cycle arrest or the activation of other proapoptotic signaling pathways is not understood and, until now, has been poorly evaluated. I believe that upcoming studies into this aspect will both provide insight into the mechanism of PARPi and help to better decipher the cellular functions and networks of these proteins.
